# A Randomized Trial Comparing 3- versus 4-Monthly Cardiac Monitoring in Patients Receiving Trastuzumab-Based Chemotherapy for Early Breast Cancer

**DOI:** 10.3390/curroncol28060427

**Published:** 2021-12-03

**Authors:** Susan Dent, Dean Fergusson, Olexiy Aseyev, Carol Stober, Gregory Pond, Arif A. Awan, Sharon F. McGee, Terry L. Ng, Demetrios Simos, Lisa Vandermeer, Deanna Saunders, John F. Hilton, Brian Hutton, Mark Clemons

**Affiliations:** 1 Division of Medical Oncology, Department of Medicine, The Ottawa Hospital, The University of Ottawa, Ottawa, ON K1H 8L6, Canada; susan.dent@duke.edu (S.D.); aawan@ohri.ca (A.A.A.); shmcgee@toh.ca (S.F.M.); teng@toh.ca (T.L.N.); jfhilton@toh.ca (J.F.H.); 2 Cancer Therapeutics Program, The Ottawa Hospital Research Institute, Ottawa, ON K1H 8L6, Canada; cstober@ohri.ca (C.S.); lvandermeer@ohri.ca (L.V.); dsaunders@ohri.ca (D.S.); 3 Clinical Epidemiology Program, The Ottawa Hospital Research Institute, The University of Ottawa, Ottawa, ON K1H 8L6, Canada; dafergusson@ohri.ca (D.F.); bhutton@ohri.ca (B.H.); 4 Thunder Bay Regional Cancer Care Northwest, Thunder Bay Regional Health Sciences Centre, Thunder Bay, ON P7B 6V4, Canada; aseyevo@tbh.net; 5 Department of Oncology, McMaster University, Hamilton, ON L8S 4L8, Canada; gpond@mcmaster.ca; 6 Stronach Regional Cancer Center, Southlake Regional Health Care Centre, Newmarket, ON L3Y 2P9, Canada; dsimos@southlakeregional.org

**Keywords:** trastuzumab, breast cancer, cardiac monitoring

## Abstract

Purpose: The optimal frequency for cardiac monitoring of left ventricular ejection fraction (LVEF) in patients receiving trastuzumab-based therapy for early breast cancer (EBC) is unknown. We conducted a randomized controlled trial comparing 3- versus 4-monthly cardiac monitoring. Patients and Method: Patients scheduled to receive trastuzumab-containing cancer therapy for EBC with normal (>53%) baseline LVEF were randomized to undergo LVEF assessments every 3 or 4 months. The primary outcome was the change in LVEF from baseline. Secondary outcomes included the rate of cardiac dysfunction (defined as a decrease in the LVEF of ≥10 percentage points, to a value <53%), delays in or discontinuation of trastuzumab therapy, and cardiology referral. Results: Of the 200 eligible and enrolled patients, 100 (50%) were randomized to 3-monthly and 100 (50%) to 4-monthly cardiac monitoring. Of these patients, 98 and 97 respectively underwent at least one cardiac scan. The estimated mean difference in LVEF from baseline was −0.94% (one-sided 95% lower bound: −2.14), which exceeded the pre-defined non-inferiority margin of −4%. There were also no significant differences between the two study arms for any of the secondary endpoints. The rate of detection of cardiac dysfunction was 16.3% (16/98) and 12.4% (12/97) in the 3- and 4-monthly arms, respectively (95% CI: 4.0 [−5.9, 13.8]). Conclusions: Cardiac monitoring every 4 months was deemed non-inferior to that every 3 months in patients with HER2-positive EBC being treated with trastuzumab-based therapy. Given its costs and inconvenience, cardiac monitoring every 4 months should be considered standard practice. Registration: NCT02696707, 18 February 2016.

## 1. Introduction

Despite the extensive global use of trastuzumab, there is minimal high-quality evidence on the optimal schedule of routine cardiac monitoring [1,2]. While well-intentioned, the high frequency of cardiac monitoring (baseline, every 3 months during treatment) recommended by the FDA in patients receiving trastuzumab-based therapy has led to increased detection of asymptomatic drops in LVEF [3], the clinical significance of which is unknown. Detection of early (asymptomatic) cardiotoxicity places patients at risk of not completing their intended HER2-targeted therapy, thus increasing the risk of cancer recurrence and death in the adjuvant setting [4]. Current position statements/guideline statements recommend LVEF evaluation every 3 [1,2,5,6,7] or 4 [8] months during trastuzumab-based therapy; however, healthcare providers are now questioning the value of cardiac monitoring in patients at low risk of cardiotoxicity—especially in the context of the global COVID-19 pandemic [9,10].

In view of this variability in practice, the current study was designed to compare two standards of care schedules (3- versus 4-monthly imaging) for cardiac monitoring of patients receiving trastuzumab-based chemotherapy for EBC. It was hypothesized that cardiac monitoring (echocardiogram/MUGA) every 4 months would not be inferior to monitoring every 3 months for the detection of changes in LVEF, and as a result, there would be no difference in the rate of detection of cardiac dysfunction.

## 2. Patients and Methods

Eligible patients with histologically confirmed HER2-positive EBC (stages I–III), with no prior history of chemotherapy, and who were scheduled to receive one year of neo/adjuvant trastuzumab-containing cancer therapy were approached for study participation by their treating oncologist at three cancer centers in Ontario, Canada. Patients had to be able to provide verbal consent through the integrated consent model [11] and have a normal LVEF (>53%) prior to initiation of trastuzumab therapy. Patients with a contraindication to receiving trastuzumab were excluded. Regulatory approval for this study was granted by the research ethics board at each participating center (OHSN-REB 20150777-01H).

### 2.1. Randomization

This study was a multi-center, two-arm, open-label, randomized non-inferiority trial. Eligible and consented patients were randomized 1:1 to either 3- or 4-monthly cardiac monitoring during trastuzumab-based therapy. While the choice of imaging modality (i.e., echocardiogram or MUGA) was at the discretion of the provider, physicians were requested to use the same imaging modality throughout the study. Assignment to treatment groups was stratified by center and chemotherapy backbone (anthracycline- vs. non-anthracycline-based). Randomization was performed using a permuted block design of variable block sizes of 4 and 6 developed by The Ottawa Methods Centre.

### 2.2. Procedures

Trastuzumab (Herceptin^®^; Genentech, San Francisco, CA, USA) dose and dosing interval were as per the standard of care. Follow-up visits during chemotherapy and trastuzumab occurred as per usual care as the study did not mandate visit schedules. Physicians could order additional cardiac evaluations if they felt it was warranted.

### 2.3. Outcomes

The primary objective was to demonstrate that cardiac monitoring (echocardiogram/MUGA) every 4 months was not inferior to that every 3 months in detecting rates of cardiac dysfunction. To evaluate this, the primary endpoint was change in LVEF throughout the course of trastuzumab-based therapy. Secondary endpoints included the frequency of detection of cardiac dysfunction (defined as a decrease of ≥10% in LVEF to below a threshold of 53% [12,13] as measured by echocardiography or MUGA), rates of delay or discontinuation of trastuzumab therapy, and referrals to cardiology. Cardiac adverse events were collected and defined according to the Common Terminology Criteria for Adverse Events (CTCAE) version 4.0 as well as study-specific questions on cardiac-related emergency room visits and hospitalizations, referral to cardiology, and changes in cardiac medications. Information on comorbidities (e.g., CAD/stroke/peripheral vascular disease, smoking, atrial fibrillation, obesity, hypertension, dyslipidemia, and diabetes) was collected at baseline for all patients. Outcome data were collected from case report forms completed by the physician when the patient was seen in clinic and after each cardiac evaluation as well as from the patient’s electronic health records.

A protocol amendment was made on 29 January 2018 to add the collection of health system utilization data using the EQ-5D-5L questionnaire [14] as well as cardiac medication and emergency room visits at baseline, following the first cardiac monitoring scan (i.e., after month 3 or 4) and at the end of the follow-up.

### 2.4. Sample Size Calculation

The sample size calculation was based on a normal LVEF >53% (mean is 61% with standard deviation of 8%). In adjuvant clinical trials, the majority of patients during trastuzumab treatment experienced a reversible decrease in LVEF of between 4 and 6%. The non-inferiority margin between groups was set as 4% (MLVEF_3m_-MLVEF_4m_ < ±4%) using a one-sided 95% confidence interval. Based on these considerations, a sample size of 87 patients in each group was required. To account for potential drop-out, the sample size was increased by approximately 10%, and recruitment of 200 participants was targeted.

### 2.5. Statistical Considerations

#### 2.5.1. Per Protocol Analysis

As we were assessing non-inferiority, the primary analysis was based on the per protocol population, which is a more conservative approach. The per protocol population (PP) consisted of all patients who consented to treatment, met all the eligibility requirements, were randomized to a treatment, and had their first cardiac scan as per the assigned allocation. If the cardiac scanning frequency changed after the initial cardiac scan, patients were still assessed in the per protocol population; however, the reasons for the change in scanning frequency were determined.

#### 2.5.2. Intention-to-Treat Analysis

A supportive analysis was performed using the ITT population, which consisted of all patients who consented to treatment, met all the eligibility requirements, and were randomized to a treatment. Additionally, secondary analyses were based on the ITT population. Patients who underwent cardiac scanning at a frequency which differed from that to which they were initially allocated were considered part of the ITT population.

#### 2.5.3. General Statistical Considerations

Baseline characteristics are presented using descriptive statistics. Outcomes of interest were estimated with two-sided confidence intervals, and statistical significance was defined at the α = 0.05 level.

#### 2.5.4. Analysis of the Primary Outcome (LVEF)

The primary outcome (LVEF) was measured at baseline and every 3 or 4 months, with the primary (and common) time point measure at 1 year. A repeated measures analysis was used to estimate the expected change in treatment effect and confidence interval, i.e., whether or not the 4-monthly group was found to be inferior by a margin of 4%. If the lower bound of the 95% one-sided confidence interval included the non-inferiority margin, the 4-monthly regimen would be deemed non-inferior to the 3-monthly regimen. As a supportive analysis, the mean difference between both treatment groups at one year of follow-up and the last measured LVEF while on trastuzumab treatment was assessed and summarized.

#### 2.5.5. Subgroup Analyses

Differences between subgroups were explored for the following a priori selected subgroups: treatment center and use of anthracycline- versus non-anthracycline-based chemotherapy.

## 3. Results

### 3.1. Patients

**Figure 1:**
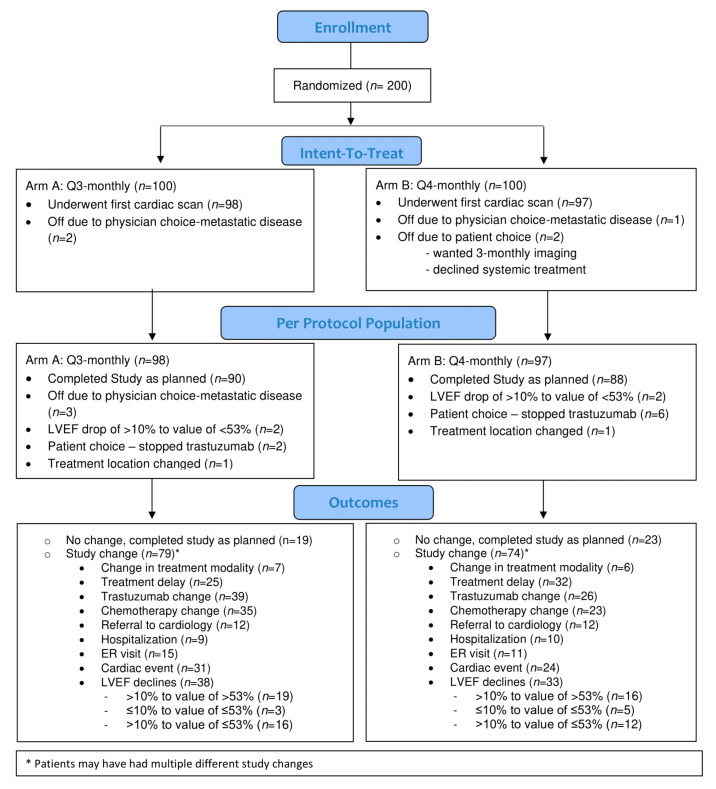
CONSORT flow diagram.

Between 6 June 2016 and 30 April 2019, 200 patients were enrolled (CONSORT diagram, Figure 1).

Of these eligible and consented patients, 100 (50%) were randomized to 3-monthly cardiac monitoring and 100 (50%) to 4-monthly imaging. The baseline characteristics of the per protocol (PP) population (i.e., all patients who consented to treatment, met all eligibility requirements, were randomized to treatment, and had their first cardiac scan as per the assigned allocation) and the intention-to-treat (ITT) population (i.e., all patients who consented to treatment, met all eligibility requirements, and were randomized) are presented in Table 1 and Supplemental Table S1, respectively.

For the PP population, the median age of patients in the 3- and 4-monthly arms was 55.7 years [IQR 47.0–62.0] and 56.0 years [IQR 47.3–62.1], respectively. Baseline LVEF (medians: 65% [IQR 60–68] vs. 64% [IQR 61–67]) and use of echocardiogram for cardiac monitoring (72.5% vs. 71.1%) were well balanced in both the 3- and 4-monthly arms (Table 2). The patient population was generally healthy, with 49 patients (50.0%) in the 3-month arm and 51 (52.6%) in the 4-month arm having baseline cardiovascular risk factors. In addition, anthracycline-based chemotherapy was received by a total of 53 (54.1%) and 50 (51.6%) patients in the 3- and 4-monthly arms, respectively. Use of cardiac medications was only collected after the protocol amendment and was well matched in the two study arms (Table 2). At the time of analysis, the median follow-up duration was 365 days for patients in both arms.

### 3.2. Primary Outcome

The median absolute change in LVEF was −8% [IQR −1, −4] in the 3-monthly arm and −6% [IQR −10, −2] in the 4-monthly arm (95% CI for the mean change between interventions: −0.6 [IQR −2.5, 1.2]) (Table 2). The mean values of LVEF over the study period are shown in Table 3. By the end of week 48, the mean (standard deviation (SD)) LVEF was 60.7 (6.2) for patients in the Q3-montly arm and 60.3 (7.0) for patients in the Q4-monthly arm. This translates to a mean (SD) change in baseline of −7.5% (6.2%) and −6.8% (6.9) for the Q3- and Q4-monthly arms, respectively. The results of the repeated measures analysis are in Supplemental Table S2 and show no interaction effect. The estimated mean difference in LVEF for patients on trastuzumab was −0.94% (one-sided 95% lower bound: −2.14), which exceeded the pre-defined non-inferiority margin of −4%.

### 3.3. Secondary Outcomes

Findings regarding the secondary outcomes are presented in Table 2. Any change in LVEF from baseline at any time during the study period was reported in a total of 79 (80.6%) and 74 (76.3%) patients in the 3- and 4-monthly arms, respectively (95% CI: 4.3 (−7.2, 15.9)). The variables used in the definition of cardiac dysfunction (i.e., decrease in LVEF of ≥10%, to a threshold of <53%) and the rate of cardiac dysfunction are shown in Table 2. The rate of detection of cardiac dysfunction was 16.3% (16/98) and 12.4% (12/97) in the 3- and 4-monthly cardiac monitoring arms, respectively (two-sided 95% CI: 4.0 (−5.9, 13.8)).

A similar number of patients experienced changes in trastuzumab administration in the 3-monthly and 4-monthly arms (14.3% vs. 10.3%, respectively), with a difference of 4.0% (95% CI = −5.2 to 13.2%). Similarly, 35 (35.7%) and 23 (23.7%) patients, respectively, had a change in chemotherapy (12.0%, 95% CI = −0.7 to 24.7%). The rates of referral to cardiology (12.2% vs. 12.4%), incidence of grade heart failure (one patient vs. none), and cardiac-related emergency room visits (one patient vs. none) were similar in the study arms (Table 2).

No interaction effect was observed based on site of recruitment (*p*-value = 0.18) or prior anthracycline use (*p*-value = 0.15) with the intervention arm. No effect due to site (*p*-value = 0.53) was observed. The mean decrease in LVEF from baseline was greater amongst patients receiving anthracycline-based chemotherapy (mean −6.5%, SD = 6.3%, across all time points) compared to non-anthracycline-based chemotherapy (mean −4.4%, SD = 6.4%; estimate = −1.52; 95% CI = −2.52 to −0.52) in the repeated measures model.

## 4. Discussion

Despite the widespread adoption of trastuzumab-based cancer therapies in the treatment of early stage HER2+ breast cancer, the optimal frequency of cardiac imaging is unknown, with most guidelines recommending either 3- or 4-monthly cardiac imaging [6,8,15]. This variability is in part driven by local resources and patient access and compliance as well as differences in the type of imaging test performed, including transthoracic echocardiography (echocardiogram) and multiple gated acquisition scan (MUGA); the chemotherapy backbone used; and the different endpoints used to define “significant cardiotoxicity” [9,16]. The current pragmatic trial permitted different cardiac imaging modalities with the use of any chemotherapy backbone. To our knowledge, there are no prospective clinical trials that have addressed the frequency of cardiac imaging for HER2-targeted therapies [4].

The current study confirmed that 4-monthly LVEF evaluation was not inferior to 3-monthly imaging. The reported incidence of cardiac dysfunction was 16.3% (16/98) and 12.4% (12/97) in the 3- and 4-monthly arms, respectively (95% CI: 4.0 [IQR −5.9, 13.8]; *p* = 0.69), which is similar to that reported in other trials [4]. Comparison with other trials is challenging as other studies have evaluated different chemotherapy regimens [17,18,19,20], used various definitions of cardiac dysfunction [12,17,21,22,23], and used different imaging modalities (e.g., MUGA or echocardiograms) [24,25]. Similarly, there were no significant differences in the secondary outcomes including changes in LVEF, delays or discontinuation of trastuzumab therapy, or referral to cardiology.

Given the increasing cost and complexity of clinical trials, the REaCT program was designed to perform practical pragmatic trials. These trials have previously evaluated surgical treatment [26], adjuvant treatment [27], use of central lines [28], supportive care [29], and palliative care (interventions) [30], but this is the first trial evaluating whether the integrated consent model is feasible with regards to ordering routine imaging modalities. Feasibility of trial design and protocol uptake was assessed through physician engagement (defined as the percentage of medical oncologists who agreed to participate in the trial compared with physicians that actually approached patients regarding the trial). For each study site, physician engagement was as follows: Ottawa, 14/16 physicians; Southlake, 4/8 physicians; and Thunder Bay, 3/5 physicians, for an overall engagement of 72% (21/29).

As with all clinical trials, there are study limitations. Firstly, it has a relatively small sample size, being performed at three Canadian cancer centers. However, the use of broad inclusion criteria meant that the study population reflected real-world practice. At the time the study was performed, there was little concurrent use of pertuzumab and trastuzumab in Ontario. Like all studies, there may be limitations on the types of patients enrolled, with physicians tending to recruit mainly young, healthy patients. There is also the limitation of the imaging techniques as only either MUGAs or echocardiograms were allowed; however, this too reflects real-world practice. This limitation would also include the fact that we did not prospectively design the study to collect data on either strain values for the imaging techniques used or NHYA data on those referred for cardiology assessment.

Future trials should address whether changes towards non-anthracycline-based chemotherapy should alter cardiac monitoring strategies, as the majority of patients are younger, at low risk of cardiac complications, and may not require such frequent monitoring. Similarly, we need to identify a priori those patients who are at increased risk of cardiac-related treatment complications. Whether this should be through novel imaging techniques or biomarker measurement remains to be elucidated [4]. Another important factor that future studies should address includes optimizing the frequency and duration of cardiac imaging after completion of trastuzumab therapy, something the current study did not evaluate.

## 5. Conclusions

Cardiac monitoring every 4 months was non-inferior to that every 3 months in early stage HER2-positive breast cancer patients being treated with trastuzumab-based therapy and should therefore be considered the standard of care.

## Figures and Tables

**Table 1:** Characteristics (per protocol population).

	Q3-Monthly	Q4-Monthly
*N*	98	97
Patient Characteristics		
**Age**		
Median [IQR] range	55.7 [47.0–62.0] 32–83	56.0 [47.3–62.1] 28–77
Mean (SD)	55.4 (11.4)	54.8 (11.3)
**Baseline LVEF**		
Median (IQR) range	65 [60–68] 54–82	64 [61–67] 54–76
Mean (SD)	64.5 (5.4)	64.0 (5.0)
Baseline modality, *N* (%) echocardiogram	71 (72.5)	69 (71.1)
CV risk factors, *N* (%) Yes	49 (50.0)	51 (52.6)
CAD stroke PVD, *N* (%) Yes	1 (1.0)	0 (0.0)
Atrial fibrillation, *N* (%) Yes	1 (1.0)	2 (2.1)
Obesity, *N* (%) Yes	10 (10.2)	15 (15.5)
**Smoking status**		
Non-smoker, *N* (%)	81 (82.7)	79 (81.4)
Current smoker	5 (5.1)	5 (5.2)
Past smoker	12 (12.2)	13 (13.4)
Hypertension, *N* (%) Yes	21 (21.4)	19 (19.6)
Diabetes, *N* (%) Yes	9 (9.2)	3 (3.1)
Angina, *N* (%) Yes	1 (1.0)	0 (0.0)
Dyslipidemia, *N* (%) Yes	12 (12.2)	7 (7.2)
Other risk factors *, *N* (%) Yes	5 (5.1)	6 (6.2)
**Treatments**		
**Chemotherapy type**		
*N* (%) Anthracycline-based	53 (54.1)	50 (51.6)
**Radiation On-Study**		
*N* (%) Yes	79 (80.6)	68 (70.1)
Median (range) dose	50 (40–92.6)	50 (40–87.1)
*N* (%) Location: Left	45 (57.0)	35 (51.5)
left	34 (43.0)	32 (47.1)
Both	0 (0.0)	1 (1.5)
**Medication information (available in only *n* = 83 patients)**	39	43
Medication, *N* (%) Yes	6 (15.4)	10 (23.3)
Aspirin, *N* (%) Yes	0 (0.0)	1 (2.3)
ACE-inhibitor, *N* (%) Yes	3 (7.7)	7 (16.3)
Beta blocker, *N* (%) Yes	3 (7.7)	0 (0.0)
Angiotensin blocker, *N* (%) Yes	1 (2.6)	3 (7.0)
CA channel antagonist, *N* (%) Yes	3 (7.7)	0 (0.0)
Diuretic, *N* (%) Yes	2 (5.1)	3 (7.0)
Statin, *N* (%) Yes	1 (2.6)	1 (2.3)
Other medications, *N* (%) Yes	0 (0.0)	1 (2.3)
**Outcomes**		
Completed study as planned		
*N* (%) Yes	90 (91.8)	88 (90.7)
Reason = LVEF	2	2
Stopped trastuzumab	2	6
Metastatic disease	3	0
Location changed	1	1

* Other risk factors include arrhythmia, mitral and aortic stenosis, myxoma, polycystic ovarian syndrome, pulmonary embolism, supraventricular tachycardia, thrombophlebitis, and triple bypass hypocholesterolemia.

**Table 2:** Outcomes (per protocol population).

		Q3-Monthly	Q4-Monthly	Diff (95% CI)
*N*		98	97	
**Primary Outcome**				
**Any change in LVEF from baseline**	*N* (%) Yes	79 (80.6)	74 (76.3)	4.3 (−7.2, 15.9)
**Absolute change in LVEF**	Median [IQR] range	−8 [−1, −4] −26 to 17	−6 [−10, −2] −33 to 10	−0.6 [−2.5, 1.2]
	Mean (SD)	−7.4 (6.5)	−6.8 (6.8)	
**Secondary Outcomes**				
**Change in LVEF**	No decline	60 (61.2)	64 (66.0)	
	Decline >10% to value of >53%	19 (19.4)	16 (16.5)	
	Decline ≤10% to value of ≤53%	3 (3.1)	5 (5.2)	
	Decline >10% to value of ≤53%	16 (16.3)	12 (12.4)	
**Cardiac dysfunction**	*N* (%) Yes	16 (16.3)	12 (12.4)	4.0 (−5.9, 13.8)
**Cardiac event**	*N* (%) Yes	31 (31.6)	24 (24.7)	6.9 (−5.7, 19.5)
Decrease in EF		6 (66.7)	10 (90.9)	
Congestive heart failure		1 (11.1)	0 ()	
Other *		2 (22.2)	1 (9.1)	
**Change in type of imaging modality**	*N* (%) Yes	7 (7.1)	6 (6.2)	1.0 (−6.0, 8.0)
**Trastuzumab**	*N* (%) Yes	14 (14.3)	10 (10.3)	4.0 (−5.2, 13.2)
Delays		9 (9.2)	2 (2.1)	7.1 (0.7, 13.5)
Reduction		3 (3.1)	1 (1.0)	2.0 (−1.9, 6.0)
Discontinuation **		2 (2.0)	7 (7.2)	−5.2 (−11.0, 0.7)
**Chemotherapy**	*N* (%) Yes	35 (35.7)	23 (23.7)	12. 0 (−0.7, 24.7)
Delay		7 (7.1)	9 (9.3)	−2.1 (−9.8, 5.6)
Reduction		12 (12.2)	6 (6.2)	6.1 (−2.0, 14.1)
Discontinuation		16 (16.3)	8 (8.3)	8.1 (−1.1, 17.2)
**Referral to cardiology**	*N* (%) Yes	12 (12.2)	12 (12.4)	−0.1 (−9.4, 9.1)
Treatment-related hospitalization ***	*N* (%) Yes	9 (9.2)	10 (10.3)	−1.1 (−9.5, 7.2)
Treatment-related ER visit ****	*N* (%) Yes	15 (15.3)	11 (11.3)	4.0 (−5.6, 13.5)

* Other refers to change in blood pressure, chest pain/shortness of breath, or irregular heartbeat. ** Reasons included decrease in EF, patient choice, timing around breast surgery for neoadjuvant patients, ER visit, or a hospitalization. *** There were no cardiac-related hospitalizations. **** There was one cardiac-related ER visit due to shortness of breath but ECG was normal.

**Table 3:** Mean (SD) LVEF over time.

Time Period	*N*	Q3-Monthly	Q4-Monthly
Week 12	97	61.2 (5.9)	
Week 16	95		61.0 (5.2)
Week 24	97	59.7 (5.7)	
Week 32	94		61.2 (6.1)
Week 36	95	60.6 (6.2)	
Week 48	93/93	60.7 (6.2)	60.3 (7.0)
Mean (95% CI) difference at week 48		0.40 (−1.48, 2.34)	
Change in LVEF from baseline			
Week 12	97	−3.3 (6.2)	
Week 16	95		−2.9 (5.3)
Week 24	97	−6.0 (6.4)	
Week 32	94		−5.0 (5.8)
Week 36	95	−6.9 (6.3)	
Week 48	93/93	−7.5 (6.2)	−6.8 (6.9)
Mean (95% CI) difference at week 48		−0.69 (−2.59, 1.21)	

## Data Availability

The data generated and/or analyzed during the current study are available from the corresponding author on request with approval from the Ottawa Health Science Network Research Ethics Board.

## Supplementary Materials

The following are available online at https://www.mdpi.com/article/10.3390/curroncol28060427/s1, Table S1: Baseline characteristics (intention-to-treat population); Table S2: Analysis of change in LVEF from baseline (per protocol population).
