# Supplementary material for: A Randomized Trial Comparing 3- versus 4-Monthly Cardiac Monitoring in Patients Receiving Trastuzumab-Based Chemotherapy for Early Breast Cancer
Source: Curr Oncol. 2021 Dec 3;28(6):5073–83. doi: 10.3390/curroncol28060427 (PMC8700071; doi:10.3390/curroncol28060427)
Supplement: Supplementary file 1 [file curroncol-28-00427-s001.zip › curroncol-1471040-supplementary.pdf]

Supplementary Materials

A Randomized Trial Comparing 3- versus 4-Monthly Cardiac Monitoring in Patients Receiving Trastuzumab-Based Chemotherapy for Early Breast Cancer

Susan Dent, Dean Fergusson, Olexiy Aseyev, Carol Stober, Gregory Pond, Arif A. Awan, Sharon F. McGee, Terry L. Ng, Demetrios Simos, Lisa Vandermeer, Deanna Saunders, John F. Hilton, Brian Hutton, and Mark Clemons, on behalf of the REaCT Investigators

Table S1. Baseline Characteristics (Intention to treat population).

		Q3-Monthly	Q4-Monthly
**N**		**100**	**100**
	**Patient Characteristics**		
**Age**	Median (IQR) range	55.8 (47.1-61.9) 32-83	56.1 (47.3-63.1) 28-77
	Mean (sd)	55.4 (11.3)	55.0 (11.3)
**Baseline LVEF**	Median (IQR) range	65 (60-68) 54-82	64 (61-67) 54-76
	Mean (sd)	64.4 (5.4)	63.9 (4.9)
**Baseline Modality**	N (%) ECHO	72 (72.0)	71 (71.0)
**CV Risk Factors**	N (%) Yes	51 (51.0)	53 (53.0)
**CAD Stroke PVD**	N (%) Yes	1 (1.0)	0 (0.0)
**Atrial Fibrillation**	N (%) Yes	1 (1.0)	2 (2.0)
**Obesity**	N (%) Yes	11 (11.0)	15 (15.0)
**Smoking Status**	N (%) Non-Smoker	82 (82.0)	80 (80.0)
	Current Smoker	5 (5.0)	7 (7.0)
	Past Smoker	13 (13.0)	13 (13.0)
**Hypertension**	N (%) Yes	22 (22.0)	20 (20.0)
**Diabetes**	N (%) Yes	9 (9.0)	3 (3.0)
**Angina**	N (%) Yes	1 (1.0)	0 (0.0)
**Dyslipidemia**	N (%) Yes	12 (12.0)	7 (7.0)
**Other Risk Factors***	N (%) Yes	5 (5.0)	6 (6.0)
	**Treatments**		
**Chemotherapy Type**	N (%) Anthracycline Based	55 (55.0)	51 (51.0)
**Radiation On-Study**	N (%) Yes	79 (79.0)	69 (69.0)
	Median (range) Dose	50 (40-92.6)	50 (40-87.1)
	N (%) Location=Left	45 (57.0)	35 (50.7)
	Right	34 (43.0)	33 (47.8)
	Both	0 (0.0)	1 (1.5)
	**Medication Information (available in only n=83 patients)**		
	N	39	44
**Medication**	N (%) Yes	6 (15.4)	11 (25.0)
**Aspirin**	N (%) Yes	0 (0.0)	1 (2.3)
**ACE-Inhibitor**	N (%) Yes	3 (7.7)	7 (15.9)
**Beta Blocker**	N (%) Yes	3 (7.7)	0 (0.0)
**Angiotensin Blocker**	N (%) Yes	1 (2.6)	4 (9.1)
**CA Channel Antagonist**	N (%) Yes	3 (7.7)	0 (0.0)

**Diuretic**	N (%) Yes	2 (5.1)	3 (6.8)
**Alpha-Blocker**	N (%) Yes	0 (0.0)	0 (0.0)
**Hydralazine**	N (%) Yes	0 (0.0)	0 (0.0)
**Clonidine**	N (%) Yes	0 (0.0)	0 (0.0)
**Spironolactone**	N (%) Yes	0 (0.0)	0 (0.0)
**Statin**	N (%) Yes	1 (2.6)	1 (2.3)
**Other Medications**	N (%) Yes	0 (0.0)	1 (2.3)
**Outcomes**			
**Completed Study as Planned**	N (%) Yes	90 (90.0)	88 (88.0)
	Reason=LVEF	2	2
	Stopped Trastuzumab	2	6
	Metastatic Disease	5	1
	Wants Cardiac Monitoring	0	1
	Declined Treatment	0	1
	Location Changed	1	1

* Other risk factors include: arrhythmia, mitral and aortic stenosis, myxoma, polycystic ovarian syndrome, pulmonary embolism, supraventricular tachycardia, thrombophlebitis and triple bypass hypocholesterolemia

Table S2. Analysis of change in LVEF From Baseline (Per protocol population).

Factor		Estimate (std error)	95% CI	p-value
Intervention Arm	Q3 vs Q4-monthly	−0.98 (0.66)		0.14
Time from Baseline Interaction	Per day	−0.017 (0.003)		<0.001
	Interaction	0.0004 (0.004)		0.92
Intervention Arm Time from Baseline	Q3 vs Q4-monthly	−0.94 (0.52)	−1.96 to 0.09	0.074
	Per day	−0.01667 (0.0020)		<0.001
